# Clinical applications for exosomes: Are we there yet?

**DOI:** 10.1111/bph.15432

**Published:** 2021-05-03

**Authors:** Dany Perocheau, Loukia Touramanidou, Sonam Gurung, Paul Gissen, Julien Baruteau

**Affiliations:** ^1^ Genetics and Genomic Medicine, Great Ormond Street Institute of Child Health University College London London UK; ^2^ Metabolic Medicine Department Great Ormond Street Hospital for Children NHS Foundation Trust London UK

**Keywords:** cancer, exosome, immunomodulation, infectious diseases, inflammation, manufacturing, therapeutics

## Abstract

Exosomes are a subset of extracellular vesicles essential for cell–cell communication in health and disease with the ability to transport nucleic acids, functional proteins and other metabolites. Their clinical use as diagnostic biomarkers and therapeutic carriers has become a major field of research over recent years, generating rapidly expanding scientific interest and financial investment. Their reduced immunogenicity compared to liposomes or viral vectors and their ability to cross major physiological barriers like the blood–brain barrier make them an appealing and innovative option as biomarkers and therapeutic agents. Here, we review the latest clinical developments of exosome biotechnology for diagnostic and therapeutic purposes, including the most recent COVID‐19‐related exosome‐based clinical trials. We present current exosome engineering strategies for optimal clinical safety and efficacy, and assess the technology developed for good manufacturing practice compliant scaling up and storage approaches along with their limitations in pharmaceutical industry.

**Abbreviations**
5‐FU: 5‐fluorouracil
EGFR: epidermal growth factor receptor
PEG: polyethylene glycol
piRNA: p‐element‐induced wimpy testis (PIWI)‐interacting RNA
siRNA: small interfering RNA
TRAIL: TNF‐related apoptosis‐inducing ligand
VEGF: vascular endothelial growth factor

## 1 INTRODUCTION

**FIGURE 1:**
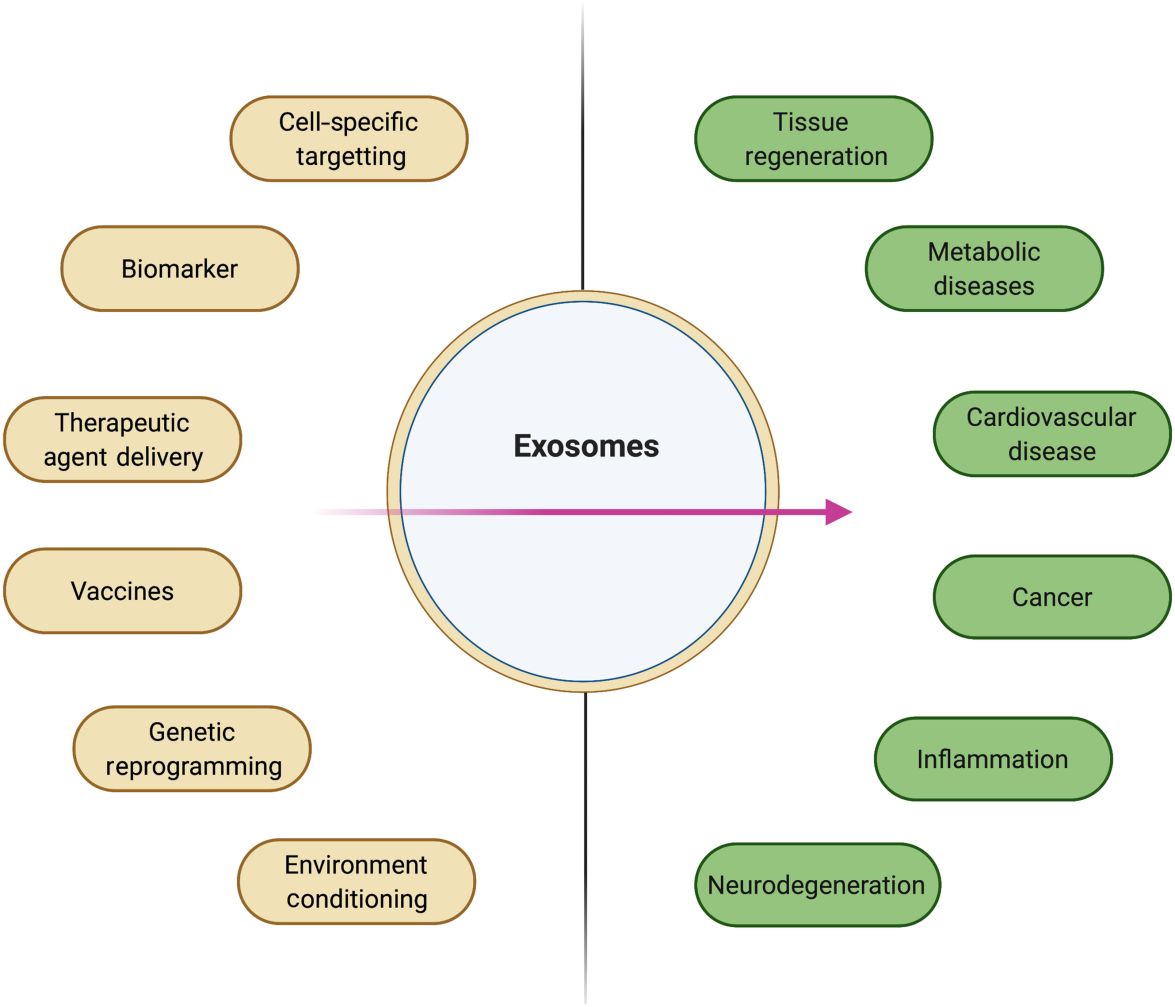
From mode of actions to therapies. Exosomes are promising players where their polymorph uses (orange) can influence their purpose (green) in clinical settings

Developing clinical applications using exosome technology has become a major field of research over the last years. Their use as diagnostic biomarkers and therapeutic carriers is generating a lot of interest and financial investment (Lin et al., 2015; Zipkin, 2019). Attesting this, the number of clinical trials involving exosomes has risen by sevenfold over the last 5 years with targeted disease areas as diverse as cancer, neurodegeneration, inflammation and immunology (NIH U.S, n.d.). Exosomes are involved in a wide range of physiological processes such as immune response (Buschow et al., 2009), tissue repair (Cui et al., 2017; Zhang et al., 2015), stem cell maintenance (Ratajczak et al., 2006) and pathological processes in cardiovascular diseases (Bang et al., 2014; Zamani et al., 2019), neurodegeneration (Howitt & Hill, 2016), cancer (Osaki & Okada, 2019), inflammation (Deng et al., 2009) and metabolism (Kalluri & LeBleu, 2020). Exosomes are released by all cells and their markers include tetraspanin family proteins (CD9, CD63 and CD81), heat shock proteins (Hsp), actin and flotillins, endosomal sorting complex required for transport proteins (Alix and TSG101) and integrins (Zhang et al., 2019). Their cargoes include DNA, mRNA miRNA, noncoding RNA, lipids, metabolites and cytoplasmic and membrane proteins involved in the regulation of cell–cell communication in both physiological and pathophysiological conditions (Colombo et al., 2014; Kalluri & LeBleu, 2020; Raposo & Stoorvogel, 2013; Sluijter et al., 2014). These components are a signature for their cell origin and their composition can alter their pharmacokinetic attributes (Kalluri & LeBleu, 2020). Exosomes are extracellular vesicles (Pan & Johnstone, 1983) generated in the cytoplasm in the multivesicular body, which is defined by the presence of intraluminal vesicles originating from inward budding into the endosomal lumen. Multivesicular bodies get transported to the plasma membrane leading to fusion with the cell surface whereby the intraluminal vesicles get secreted as exosomes (Colombo et al., 2014). Extracellular vesicles are classified into three groups based on their sizes:‐ exosomes (40–160 nm) (Kalluri & LeBleu, 2020), microvesicles (100–1000 nm) and apoptotic bodies (>1000 nm) (Andaloussi et al., 2013; Pegtel & Gould, 2019). The size and marker overlap between exosomes and microvesicles, and make their differentiation complex (Corso et al., 2017; Doyle & Wang, 2019). However, the International Society for Extracellular Vesicles (ISEV) proposes a series of references to characterise exosomes based on their function or composition (Théry et al., 2018). Their reduced immunogenicity compared to liposomes or viral vectors, and their ability to cross major physiological barriers like the blood–brain barrier make them an attractive and innovative option as biomarkers and therapeutic agents (Figure 1). Indeed exosomes, which are present in a wide range of body fluids, could be ideal biomarkers particularly for early detection of diseases. To support translation and ever‐expanding portfolio of clinical trials, various strategies are being developed to optimise cargo loading, improve cell type targeting and increase exosome uptake. Good manufacturing practice of exosomes requires robust scaling up and storage processes (Whitford & Guterstam, 2019).

Here, we review the latest clinical developments of exosome biotechnology for diagnostic and therapeutic purposes, including the most recent COVID‐19‐related exosome‐based clinical trials. We present current exosome engineering strategies for optimal clinical safety and efficacy and assess the technology developed for good manufacturing practice compliant scaling up and storage approaches along with their limitations.

## 2 EXOSOMES AS BIOMARKERS

Exosomes released in pathophysiological conditions such as inflammation, neurodegeneration, immune response, cancer, cell death or angiogenesis contain constitutive components, including transmembrane proteins or nucleic acids that can act as biomarkers for clinical diagnosis, staging disease severity or assessing therapeutic response (Table 1) (Hardy et al., 2019; Howitt & Hill, 2016; Jiang et al., 2016; Kitai et al., 2017; Waldenström & Ronquist, 2014; Wu et al., 2016).

**TABLE 1:** Clinical trials investigating exosomes as biomarkers (from https://clinicaltrials.gov)

Medical speciality	Condition	Year of initiation	Application	Sponsor	Status	Clinical trial number
Cancers	Breast cancer	2011	Theranostic	Jenkins Cancer Center, United States	Withdrawn	NCT01344109
	Gastric cancer	2013	Diagnostic and prognostic	Hospital Miguel Servet, Saragossa, Spain	Unknown	NCT01860118
	Prostate cancer	2014	Diagnostic	Exosome Diagnostics, Inc	Completed	NCT02702856
	Metastatic melanoma	2014	Theranostic	University Hospital, Nice, France	Unknown	NCT02310451
	Pancreatic cancer	2015	Diagnostic	Memorial Sloan Kettering Cancer Center, United States	Active, not recruiting	NCT02393703
	Oropharyngeal cancer	2015	Diagnostic	New Mexico Cancer Care Alliance, United States	Recruiting	NCT02147418
	Thyroid cancer	2016	Prognostic	National Taiwan University Hospital, Taiwan	Active, not recruiting	NCT02862470
	Non‐small cell lung cancer (NSCLC)	2016	Diagnostic	Xinqiao Hospital of Chongqing, China	Unknown	NCT02890849
	Non‐small cell lung cancer (NSCLC)	2016	Diagnostic	Xinqiao Hospital of Chongqing, China	Unknown	NCT02869685
	Lung cancer	2017	Diagnostic	Wuhan Union Hospital, China	Recruiting	NCT03830619
	Pancreatic ductal adenocarcinoma (PDAC)	2017	Diagnostic	University Hospital, Bordeaux, France	Completed	NCT03032913
	Cholangiocarcinoma, benign biliary stricture	2017	Diagnostic	The Second Hospital of Nanjing Medical University, China	Unknown	NCT03102268
	Lung metastases, osteosarcoma	2017	Diagnostic and prognostic	Ruijin Hospital Shanghai Jiao Tong University School of medicine, Shanghai, China	Recruiting	NCT03108677
	Sarcoma	2018	Prognostic	Centre Georges Francois Leclerc, Dijon, France	Recruiting	NCT03800121
	High grade serous carcinoma ovarian cancer	2018	Diagnostic and prognostic	Peking Union Medical College Hospital, China	Recruiting	NCT03738319
	Early lung cancer	2018	Diagnostic	Second Affiliated Hospital of Soochow University, China	Unknown	NCT03542253
	Pancreatic cancer	2018	Diagnostic and prognostic	The Affiliated Nanjing Drum Tower Hospital of Nanjing University Medical School, China	Recruiting	NCT03821909
	Gallbladder carcinoma	2018	Prognostic	Xinhua Hospital Shanghai, China	Recruiting	NCT03581435
	Rectal cancer	2018	Prognostic	University of Kansas Medical Center, United States	Recruiting	NCT03874559
	Bone metastases	2018	Theranostic	Istituto Ortopedico Rizzoli, Italy	Recruiting	NCT03895216
	Thyroid cancer	2018	Prognostic	National Taiwan University Hospital, Taiwan	Active, not recruiting	NCT03488134
	Obstructive sleep apnoea syndrome, cancer	2019	Diagnostic	University Hospital, Angers, France	Recruiting	NCT03811600
	Breast cancer, leptomeningeal metastasis	2019	Diagnostic	Centre Oscar Lambret, Lille, France	Not yet recruiting	NCT03974204
	Prostate cancer	2019	Prognostic	Imperial College London, London, United Kingdom	Recruiting	NCT04167722
	HER2‐positive breast cancer	2019	Diagnostic	King's College London, London, United Kingdom	Recruiting	NCT04288141
	Rectal neoplasm malignant carcinoma	2019	Prognostic	Fudan University, China	Recruiting	NCT04227886
	Pulmonary nodules	2019	Diagnostic	Shanghai Chest Hospital, China	Recruiting	NCT04182893
	Clear cell renal cell carcinoma	2020	Diagnostic	University Hospital, Saint Etienne, France	Recruiting	NCT04053855
	Colorectal cancer	2020	Diagnostic and prognostic	University Hospital, Reims, France	Not yet recruiting	NCT04394572
	Non‐small cell lung cancer (NSCLC)	2020	Prognostic	Fudan University, China	Recruiting	NCT04427475
	Prostate cancer	2020	Diagnostic	Chesapeake Urology Research Associates, Baltimore, United States	Recruiting	NCT04357717
	Prostate cancer	2016	Diagnostic	University Hospital Inselspital, Switzerland	Completed	NCT03034265
Cardiovascular diseases	Hemodynamic instability	2017	Diagnostic	Taipei Tzu Chi Hospital, Buddhist Tzu Chi Medical Foundation, Taipei, Taiwan	Active, not recruiting	NCT03267160
	Atrial fibrillation	2018	Diagnostic	Sheba Medical Center, Ramat Gan, Israel	Recruiting	NCT03478410
	Myocardial infarction	2019	Diagnostic	Xinhua Hospital, Shanghai Jiao Tong University School of Medicine, China	Not yet recruiting	NCT04127591
	Prehypertension	2020	Diagnostic	Rockefeller University, United States	Recruiting	NCT04142138
Obstetrics & gynaecology	Endometrial fluid collection	2016	Diagnostic	IVI Valencia, Spain	Unknown	NCT02797834
	Oocyte maturation	2020	Prognostic	Chinese University of Hong Kong, Hong Kong	Not yet recruiting	NCT04382872
	Preeclampsia	2016	Diagnostic	Cairo University, Cairo	Completed	NCT03562715
	Preeclampsia	2020	Diagnostic	University of Alabama, Birmingham, United States	Not yet recruiting	NCT04154332
	Normal cellular metabolism	2016	Diagnostic	Mayo Clinic in Rochester, United States	Active, not recruiting	NCT02748369
Healthy	Healthy	2016	Diagnostic	University of Southern Denmark, Denmark	Completed	NCT02823613
	Healthy	2019	Diagnostic	August Krogh Building, Denmark	Recruiting	NCT03700515
Neurodegenerative diseases	Parkinson's disease	2013	Diagnostic	University of Alabama, Birmingham, United States	Completed	NCT01860118
Miscellaneous	Sepsis	2016	Prognostic	Jinling Hospital, China	Unknown	NCT02957279
	Diabetic retinopathy	2018	Prognostic	Shanghai General Hospital, Shanghai Jiao Tong University School of Medicine, Shanghai	Not yet recruiting	NCT03264976
	Kidney transplantation	2018	Theranostic	University Hospital, Bordeaux, France	Completed	NCT03503461
	Thyroid diseases, heart failure	2019	Prognostic	National Taiwan University Hospital, Taiwan	Recruiting	NCT03984006
	Postoperative delirium, general anaesthesia, circadian rhythm disorders	2020	Diagnostic	Shengjing Hospital, China	Not yet recruiting	NCT04421872
Infectious diseases	COVID‐19	2020	Theranostic biomarker	Larkin Community Hospital, Miami, Florida, United States	Recruiting	NCT04384445
				GENKOK, Kayseri, Melikgazi, Turke	Active, not recruiting	NCT04389385
				Medical Centre Dinasty Samara, Russian Federation	Enrolling by invitation	NCT04602442
				Cedars‐Sinai Medical Center Los Angeles, California, United States	Recruiting	NCT04623671
				Ruijin Hospital Shanghai Jiao Tong University School of Medicine	Completed	NCT04276987

Some of the tetraspanins, such as CD9, CD63, CD81 and CD151, often found at the exosomal membranes (Hoshino et al., 2015; Thery et al., 2002) can be used as screening tools in health and disease. For example, increased CD81 correlates with fibrosis progression and has been proposed as a diagnostic biomarker for complications in viral hepatitis C (Logozzi et al., 2009; Welker et al., 2012).

The secretion of extracellular vesicles from different types of tumour cells is a significant method of conditioning and altering the tumour microenvironment by malignant cells (Harris et al., 2015). Although exosomes are produced by most cell types, observations support the increased secretion of exosomes under pathological conditions, such as cancer (Ohno et al., 2013). Proteomic analysis of those exosomes secreted under various physiological and pathological conditions has shown significant changes in protein expression (Duijvesz et al., 2013; Lee et al., 2016). Proteins from various cancers are also involved in similar biological processes and functions. A study performed gene ontology analysis on a variety of differentially expressed proteins derived from exosomes. Results suggested that those proteins were involved in similar biological mechanisms, such as cell adhesion, migration and transport (Sherman & Lempicki, 2009). Contribution of exosomal proteins to angiogenesis, metastasis, tumour formation and disease makes them appealing biomarkers for diagnosis or prognosis applications in cancer, for example, lysosomal‐associated membrane protein 3 (LAMP‐3; also known as CD63) in ovarian cancer, lung cancer and melanoma (Hurwitz et al., 2016; Pols & Klumperman, 2009), epidermal growth factor receptor (EGFR) in glioblastoma (Skog et al., 2008), proteoglycan glypican‐1 in early stage of pancreatic cancer (Melo et al., 2015) and proteins associated with the EGFR pathway, such as retinoic acid‐induced protein 3, Gs‐α subunit and resistin in bladder cancer and kidney disorders (Smalley et al., 2008; Zhou et al., 2008).

In neurodegeneration, exosomal aggregation‐prone protein amyloid‐β peptide (amyloid β) and exosome‐associated tau phosphorylated at Thr‐181 are established diagnostic biomarkers for Alzheimer's disease (Rajendran et al., 2006; Saman et al., 2012). Autolysosomal proteins like cathepsin D, LAMP or α‐synuclein are proposed diagnostic markers for early‐stage Parkinson's disease (Alvarez‐Erviti, Couch, Richardson, et al., 2011; Alvarez‐Erviti, Seow, Schapira, et al., 2011; Alvarez‐Erviti, Seow, Yin, et al., 2011; Goetzl et al., 2015; Lemprière, 2020). Neuronal exosomal α‐synuclein, identified as the major protein of the neuropathological hallmark of idiopathic Parkinson's disease, has been reported to be three to five times higher in Parkinson's disease patients with early and advanced forms of the disease, respectively (Alvarez‐Erviti, Couch, Richardson, et al., 2011; Alvarez‐Erviti, Seow, Schapira, et al., 2011), compared to healthy individuals. In contrast, no significant difference in total α‐synuclein concentration between early and advanced Parkinson's disease patients were found (Niu et al., 2020), highlighting the exosomal counterpart to be a more sensitive diagnostic and severity scoring tool.

Exosomes also incorporate nucleic acids such as messenger RNA (mRNA), micro‐RNA (miRNA), small interfering RNA (siRNA) and p‐element‐induced wimpy testis (PIWI)‐interacting RNA (piRNA) that display mutations in the host cell (Han et al., 2017; Shtam et al., 2013; Thakur et al., 2014; Valadi et al., 2007). The early detection of a tumour is crucial for successful treatment. In addition to tissue biopsy‐based diagnosis, investigation of circulating miRNA is an expanding field in biomarker research as miRNA profiling can inform about diagnosis, prognosis, chemosensitivity and therapeutic response. miRNAs are easily accessible in biological fluids through less invasive “liquid biopsy.” Various exosomal miRNAs are being developed as noninvasive diagnostic biomarkers, such as miRNA‐21 for oesophageal squamous cell carcinoma (Ogata‐Kawata et al., 2014; Tanaka et al., 2013), miRNA‐139‐5p, miRNA‐378a, miRNA‐379 and miRNA‐200‐5p for lung carcinoma (Cazzoli et al., 2013) and miRNA‐574‐3p, miRNA‐141‐5p and miRNA‐21‐5p for prostate cancer (Thind & Wilson, 2016). Exosomal miRNAs play a direct role in influencing the cancer pathophysiology (Sharma, 2018). Several studies have focused their analysis on circulating miRNA from enriched exosomes. A recent study showed that exosomal miR‐141 was progressively increased in prostatic hypertrophy, localised prostate cancer and metastatic disease, suggesting a potential diagnostic or prognostic role (Li et al., 2016). A different project compared the performance of whole blood miRNA with exosomal miRNA analysis. The results presented that miR‐375 derived from whole plasma could differentiate pancreatic cancer from benign prostatic hyperplasia patients, while exosomal miR‐200c‐3p and miR‐21‐5p were better discriminators and Let‐7a‐5p miRNA in exosomes could distinguish pancreatic cancer patients with different severity scores (Endzeliņš et al., 2017). miRNA‐375 levels in exosomes from squamous cells of carcinoma patients determine the progression from local inflammation to carcinoma and are potential early‐stage biomarkers for oral carcinoma (Shi et al., 2015). Exosomal miRNA‐20a‐5p, miRNA‐24‐3p, miRNA106a‐5p, miRNA‐891a and miRNA‐1908 in nasopharyngeal carcinoma affect both cell differentiation and expansion by down‐regulating the mitogen‐activated protein kinase‐1 (MARK1) signalling pathway, hence their prognostic application (Ye et al., 2014). Similarly, exosomal mRNAs can be used as diagnostic biomarkers in body fluids reducing the impact and cost of surgical biopsies (Rabinowits et al., 2009; Taylor & Gercel‐Taylor, 2008). For example, saliva‐derived exosomes (Lau et al., 2013), human amniotic fluid‐derived exosomes and urine‐derived exosomes (Keller et al., 2007) manage to pack a high number of mRNAs and are used for the diagnosis of pancreatic cancer and prenatal diagnosis of renal failure, reducing the need for invasive biopsies. In 2016, a study compared the urine exosomal mRNA gene expression in 499 prostate cancer patients present with high prostate‐specific antigen (PSA), a hallmark glycoprotein enzyme used as diagnostic screening tool for prostate cancer. The subsequent prognostic score was then validated in 1064 patients. In 255 men urine exosome, gene expression assay correlated with histological discrimination and staging severity. Results showed that in approximately one third of patients, biopsy could have been avoided (McKiernan et al., 2016). In addition, piRNAs play a crucial role in transposon silencing, epigenetic regulation, genome rearrangement, germ stem‐cell maintenance and oncogenesis (Han et al., 2017; Yu et al., 2019). piRNA expression varies significantly across different somatic tissues. In cancer, different piRNA expression profiles differentiate healthy and tumour tissues (Pols & Klumperman, 2009) with clinical relevance (Siddiqi & Matushansky, 2012), promoting their use as cancer‐specific biomarkers (Yu et al., 2019). Studies revealed that numerous piRNAs have been involved in cancer development; however, only a small number of piRNAs have been found to be expressed in somatic tissues. Those piRNAs are involved in cancer cell proliferation, apoptosis, metastasis and invasion and could be used as prognostic and diagnostic markers in cancer development (Martinez et al., 2015). Various studies have also showed that piRNAs could be valuable markers for cancer metastasis, for example, piR‐4987 in lymph node metastasis (Huang et al., 2013), piR‐932 and PIWIL2 in metastasis of breast cancer (Zhang et al., 2013) and piR‐32051, piR‐39894, and piR‐43607 in clear cell renal cell carcinoma metastasis, late clinical stage and poor cancer‐specific survival (Fu et al., 2015). Recent publications have questioned whether some RNA detected in exosome preparations could be derived from culture media, particularly when using sera, following the most accepted purification method based on ultracentrifugation (Tosar et al., 2017; Wei et al., 2016).

## 3 EXOSOMES AS THERAPEUTICS

While the vast majority of ongoing exosome‐based clinical trials aims at identifying diagnostic or prognostic biomarkers (Table 1), a rapidly increasing number of trials are also investigating exosomes as therapeutic agents in a wide range of diseases including cancer, immunomodulation, neurodegeneration and infectious diseases. Mesenchymal stem cells (MSCs), dendritic cells (DCs) and even autologous tumour cells are the main sources for therapeutic exosomes in either their naïve (i.e. unmodified) or engineered form (Table 2).

**TABLE 2:** Clinical trials investigating exosomes as therapeutics (from https://clinicaltrials.gov); abbreviations: DC, dendritic cells; GVHD, graft versus host disease; MSCs , mesenchymal stem cells.

Medical specialty	Condition	Phase	Year of initiation	Origin	Therapeutic cargo	Outcome	Clinical trial number	Reference
Cancer	Vaccination against non‐small cell lung tumours	II	2010	DCs	mCTX, tumour antigen	Significant NK cell activation, no specific T cell response against tumour cells	NCT01159288	(Besse et al., 2016)
	Recurrent malignant glioma	I	2012	Tumour cells	IGF1R anti‐sense molecule	IGFIR downregulation ≤10%	NCT01550523	(Andrews et al., 2001)
	Metastatic pancreas cancer with KrasG12D mutation	I	2020	MSCs	KrasG12D siRNA	Ongoing	NCT03608631	(Kamerkar et al., 2017)
Neurology	Acute ischemic stroke	I/II	2019	MSCs	miR‐124	Unknown	NCT03384433	(Yang, Zhang, et al., 2017)
	Alzheimer's disease	I/II	2020	MSCs	Naïve	Ongoing	NCT04388982	
Ophtalmology	Refractory macular holes	I	2017	MSCs	Naïve	Unknown	NCT03437759	(Yu et al., 2020)
	Dry eye in patients with cGVHD	I/II	2020	MSCs	Naïve	Ongoing	NCT04213248	(Lai et al., 2018)
Miscellaneous	Type I diabetes mellitus	II/III	2014	MSCs	Naïve	Amelioration of inflammatory immune reaction and overall kidney function	NCT02138331	(Nassar et al., 2016; Zhao et al., 2012)
	Effects on coagulation and platelets function	n/a	2015	Red blood cells	Naïve	Unknown	NCT02594345	
	Cutaneous wound healing	I	2015	Plasma	Naïve	Unknown	NCT02565264	
	Multiple organ dysfunction post type A aortic dissection	n/a	2020	MSCs	Naïve	Ongoing	NCT04356300	(Harrell et al., 2020)
	Dystrophic epidermolysis bullosa	I/II	2020	MSCs	COL7 and COL7A1 mRNA	Ongoing	NCT04173650	
	Periodontitis	I	2020	Adipose stem cells	Naïve	Ongoing	NCT04270006	(Mohammed et al., 2018)
Infectious diseases	COVID‐19	I	2020	Covid‐19 T cell	Naïve	Ongoing	NCT04389385	
				Allogenic adipose MSCs			NCT04276987	
				MSCs			NCT04313647	
				MSCs			NCT04602442	
				MSCs			NCT04491240	
				Amniotic stem and epithelial cells			NCT04657406	

### 3.1 Cancer therapies

Different exosome‐based approaches can be used for cancer therapies like oncogene inhibition. For example, the phase I trial (NCT03608631) sponsored by the M.D. Anderson Cancer (Texas, USA) investigates the use of mesenchymal stem cells‐derived exosomes for the treatment of stage IV pancreatic cancer patients with the presence of KrasG12D mutation where patients are injected with KrasG12D‐specific siRNA‐loaded exosomes targeting the oncogenic *KRAS* gene thereby reducing its expression in pancreatic tumours (Kamerkar et al., 2017). The immunotherapy approach has also been trialled in patients with unresectable non‐small cell lung cancer in 2015 (NCT01159288) by using dendritic cells‐derived exosomes loaded with tumour antigens (Besse et al., 2016). No specific T cell response against cancer cells expressing the antigen of interest was observed, although a significant increase in NK cell activation in some patients was reported. Unfortunately, the primary endpoint of 50% nonprogressors was not met and the trial was terminated. Tumour cells are also a promising source of exosomes in cancer therapy due to their tropism and their ability to induce a specific inflammatory response. Using the patient's own tumoural cells as manufacturing cell line has the advantage of preventing neutralisation by innate immunity. A phase I trial (NCT01550523) used autologous glioma cells pretreated with insulin‐like growth factor I receptor (ILF1R) anti‐sense molecule targeting the tumour's tyrosine kinase cell surface receptors in the aim of inhibiting tumourigenesis (Andrews et al., 2001). No results have been disclosed yet.

### 3.2 Anti‐inflammation/immunomodulation therapies

Exosomes are promising delivery agents to treat inflammatory disorders due to their low immunogenicity, intrinsic anti‐inflammatory properties and drug delivery potential. For instance, mesenchymal stem cell‐derived exosomes were administered to patients with graft versus host disease and allowed to reduce the pro‐inflammatory cytokine response (Kordelas et al., 2014) (NCT04213248). This also led to ongoing trials for the treatment of patients with type I diabetes mellitus (NCT02138331) and macular degenerations (NCT03437759) using mesenchymal stem cells‐derived exosomes for their immunomodulation properties. Mesenchymal stem cells are the most common source of therapeutic exosomes particularly for regenerative medicine and immunomodulation. The phase I trial (NCT02138331) led by Nassar et al. also using mesenchymal stem cell‐derived exosomes from umbilical cord blood investigated their effect on β‐cell mass in type 1 diabetes mellitus. Cord blood‐derived multipotent stem cells have shown successful modulation of the autoimmune response against β‐cells by increasing the number of specific regulatory T cells (Treg) lymphocytes, thereby restoring the Th1/Th2 immune balance (Nassar et al., 2016; Zhao et al., 2012). No results have been disclosed yet.

### 3.3 Neurological diseases

Exosomes have the advantages of being able to cross the blood–brain barrier, a critical step for brain‐targeted therapies. This property has been exploited in diseases such as Parkinson (Haney et al., 2015) or ischemic stroke. Recently, studies have shown that nanoparticles (Liu et al., 2013; Lv et al., 2018) but also exosomes can be used as a therapeutic agent for the treatment of ischemic stroke where an engineered c(RGDyK)‐conjugated exosomes were able to target the lesion of the ischemic brain following intravenous injections (Tian et al., 2018). Reperfusion can be the only option to reverse brain damage following a stroke, but this can induce an inflammatory reaction potentially causing further damage. This is how Dong et al. (2019) suggested the use of neutrophil membrane‐derived vesicles loaded with resolvin D2, acting as anti‐inflammatory agent and specifically delivered to the brain and in particular to a stroke lesion.

### 3.4 Infectious diseases

Exosome‐based technologies to generate vaccines have been exploited for years (Devhare & Ray, 2017). Exosomes have been explored as a platform for vaccination, by delivering disease‐associated antigens. This approach was tested by delivering hepatitis C‐associated antigens (Desjardins et al., 2009) and extended to the treatment of infectious diseases by using extracellular vesicles originating from infected cells (Lener, Gimona, Aigner, Börger, Buzas, Camussi, Chaput, Chatterjee, Court, Portillo, & O'Driscoll, 2015a). In fact, exosomes derived from dendritic cells and primed with antigens against *Toxoplasma gondii* successfully mediated a protective immune response (Beauvillain et al., 2009). This fundamental approach was also tested *in vitro* against the SARS‐CoV‐2 coronavirus using exosomes loaded with the virus spike S protein (Kuate et al., 2007), providing an insight into the potential use of exosomes for vaccination against SARS‐CoV‐2.

Interestingly, various exosome‐based studies and clinical trials associated with COVID‐19 have been initiated. These trials aim to treat severe acute respiratory complications associated with COVID‐19. They include trials (NCT04276987, NCT04313647) built on previous studies highlighting the immunomodulatory role of adipose tissue‐derived mesenchymal stem cells and its secretome for the treatment of pulmonary injuries (Deffune et al., 2020) (Bari et al., 2020; Leng et al., 2020). Another trial (NCT04389385) is assessing the effect of exosomes derived from T‐cells activated against COVID‐19, delivered via an aerosol. The T‐cells are isolated from donors, activated and expanded *in vitro* by exposure to viral peptide fragments to stimulate the production of exosomes enriched with therapeutic mediators such as interferon‐γ. Neither safety nor efficacy results are available for these ongoing trials. An acellular product under the affiliation of Organicell™ flow is being tested in recent trials (NCT04602442, NCT04657406, NCT04491240). It is derived from human amniotic fluid, highly enriched in exosomes and consisting of various growth factors, pro‐inflammatory cytokines potentially able to act as a suppressor of the cytokine storm observed in COVID‐19 patients.

## 4 EXOSOME ENGINEERING

**FIGURE 2:**
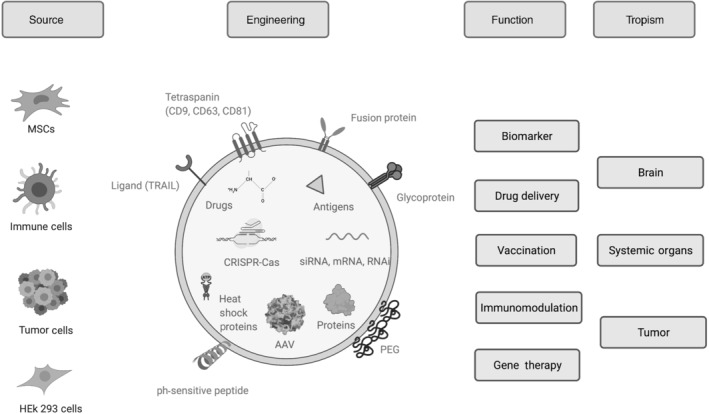
Engineering strategies to refine a specific function and/or tropism to exosomes. Depending on their parent cell line, exosomes can express intrinsic ligands or can be engineered to express specific targeting ligands, stimuli response peptide, fusion protein, immune‐evasive components, or viral glycoproteins. Their cargos can vary from small‐sized genetic material such as noncoding RNAs to components as large as AAV. Exosome engineering can enable specific targeting of the central nervous system, systemic organs, or tumours. AAV: adeno‐associated vector, HEK: human embryonic kidney, MSC: mesenchymal stem cells, PEG: polyethylene glycol, TRAIL: TNF‐related apoptosis‐inducing ligand

Exosomes are being widely investigated as immunomodulators or therapeutic cargo vehicles. This intense research field is continuously refining engineering strategies to optimise efficacy and/or delivery. A specific targeting of the recipient cell is paramount to adequately deliver exosome content (Horibe et al., 2018). This is mediated by the surface composition of the exosome acting as a signature for the uptake by the recipient cell type (Hazan‐Halevy et al., 2015; Sancho‐Albero et al., 2019). When reaching the target cell, exosomes can either trigger signalling (Guan et al., 2014) by directly interacting with extracellular receptors or release their cargo after fusion with the plasma membrane or internalisation (Mulcahy et al., 2014). A wide range of engineering strategies have therefore been developed from enhancing exosome uptake to optimising cargo but also showing the importance of the right origin of the exosome‐producing cells (Figure 2).

### 4.1 Enhancing target cell uptake

For optimal safety and efficacy, targeting specifically the recipient cell and minimising off‐target effect is essential. Exosome composition can be modified using native, exogenous, or engineered ligands like membrane proteins involved in cell–cell communication or endocytosis. Improvement in cell targeting increases the uptake of the therapeutic agent.

One of the strategies to enhance cell targeting is overexpression of specific viral proteins in exosomes. For example, Ruiss et al. (2011) overexpressed gp350, the main envelope protein of Epstein Barr virus enhancing β‐cell tropism. Similarly, HEK 293‐derived exosomes can be pseudotyped to overexpress the therapeutic protein of interest fused to the viral envelope protein vesicular stomatitis virus glycoprotein. This fusion with the vesicular stomatitis virus glycoprotein enhances loading of the therapeutic protein in exosomes without affecting the exosome size, thereby allowing increased delivery of cargo to the target cells (Meyer et al., 2017). Similarly, mouse dendritic cell‐derived exosomes, used for their low immunogenicity, were altered to overexpress an exosomal membrane protein Lamp2b fused to a tumour‐homing peptide iRGD (CRGDK/RGPD/EC). The high specificity of the iRGD peptide to α_v_ integrin resulted in approximately threefold increase *in vitro* uptake of exosomes by breast cancer cells (Tian et al., 2014). In a similar approach, Lamp2b was fused with the rabies virus glycoprotein, a neuron‐specific peptide to deliver an siRNA against beta‐secretase 1 (BACE1), a precursor for Alzheimer's disease. This led to BACE1 reduction in neurons, microglia and oligodendrocytes after systemic injection *in vivo* (Alvarez‐Erviti, Seow, Yin, et al., 2011). Cardiosphere‐derived exosomes were exploited in a similar approach by ligating Lamp2b with cardiomyocyte specific protein to mediate reduction in cardiomyocyte apoptosis and stimulate its proliferation (Mentkowski & Lang, 2019).

The pH of tumour microenvironment plays a key role in the exosome release and uptake by the cell (Parolini et al., 2009). Incorporation of pH‐sensitive peptides such as the alpha‐galactosidase A protein can be used to enhance membrane fusion. Alpha‐galactosidase A protein creates an α‐helix formation upon endocytosis and subsequent pH reduction (Nakase & Futaki, 2015). Similarly, exosome membrane while coupled with 3‐(diethylamino) propylamine results in membrane disruption in an acidic environment resulting in enhanced cytosolic release of the cargo (Lee, Park, et al., 2019).

Extracellular vesicles including exosomes are being targeted by plasma proteins making them prone to rapid clearance during circulation. Polyethylene glycol (PEG) adjunction to the outer membrane of exosomes can act as a shield to protect vesicles from clearance (Antimisiaris et al., 2018). However, this can hinder interactions between target cells and exosomes. To counteract this, PEG‐conjugated antibodies or “nanobodies” against EGFR are added onto the exosomal membrane increasing the circulation time and binding to EGFR overexpressing tumour cells (Kooijmans et al., 2016).

### 4.2 Optimising cargoes

Based on their natural capacity to transfer cargo such as proteins or nucleic acids, exosomes can be engineered as a therapeutic vehicle. However, mechanisms for loading such cargoes into exosomes are challenging. This is why recent efforts are being developed to maximise exosomal cargo loading and cargo refinement for increased therapeutic benefit. This can be achieved by creating exosome scaffolds like the prostaglandin F2 receptor negative regulator protein and the brain acid soluble protein 1 (Codiak Ltd., 2021) or by using the late domain pathway via the NEDD4 family‐interacting (Ndfip) protein resulting in the ubiquitination and loading of the protein into exosomes (Sterzenbach et al., 2017). Evox Therapeutics have recently developed engineered exosomes using proprietary exosomes producing cell lines for the delivery of a functional protein for the treatment of Niemann–Pick type C (Zipkin, 2019) but also argininosuccinic aciduria (Evox Therapeutics, 2021; Sonam Gurung, American Society of Gene and Cell Therapy, 2021).

Similarly, genetic materials such as miRNAs are naturally present in exosomes but at levels unlikely to exert detectable effects (Chevillet et al., 2014). This is why strategies have been developed to overexpress them in exosomes to mediate posttranscriptional regulation in recipient cells. For example, this involves miRNA‐126 acting as miRNA mimic to supress the PTEN/PI3K/AKT signalling pathway involved in cancer proliferation and migration (Nie et al., 2020). In another application in cancer, exosomes are used as specific drug vehicle delivering doxorubicin, paclitaxel or curcumin with a better efficacy and reduced off‐target toxicity compared to treatment with drug alone (György et al., 2015; Mentkowski et al., 2018). To tackle chemotherapy resistance, exosomes are used for co‐delivery of chemotherapeutic drug and chemoresistance inhibitors using miRNA inhibitors to modulate the expression of tumour suppressor genes. For instance, exosomes loaded with 5‐fluorouracil (5‐FU) against colorectal cancer and miR‐21 inhibitor (miR‐21‐i) successfully reduced miR‐21‐mediated 5‐FU resistance in 5‐FU‐resistant colorectal cancer cell line (Liang et al., 2020). Similar technologies can be developed to deliver other genetic materials such as siRNAs which have therapeutic applications in gene silencing or the CRISPR‐Cas gene editing systems. This is of particular interest for the treatment of neurological diseases due to the exosome's ability to cross the blood–brain barrier. Exosome‐mediated delivery of siRNA against BACE1 or the vascular endothelial growth factor (VEGF) resulted, respectively, in a strong reduction of BACE1 (Alvarez‐Erviti, Seow, Yin, et al., 2011) or VEGF (Yang, Fogarty, et al., 2017) reduction in the mouse brain (Alvarez‐Erviti, Seow, Yin, et al., 2011). CRISPR‐Cas systems can also be delivered using exosomes instead of viral vectors where safety and logistical challenges to package such large cargoes have created limitations for wider use (Kim et al., 2017; Knott & Doudna, 2018).

The ability of exosomes in protecting their content against degradation is another advantage for some cargoes such as adeno‐associated vector‐mediated vectors. This system protects adeno‐associated vectors against specific capsid humoral immunity in preimmunised patients, an increasing issue in adeno‐associated vector gene therapy (Perocheau et al., 2019). A subpopulation of adeno‐associated vectors originally from the media of manufacturing cells is physiologically encapsulated into exosomes creating exosomes‐associated adeno‐associated vectors (exo‐adeno‐associated vectors) (György & Maguire, 2018). Exosomes‐associated adeno‐associated vectors enable successful transduction *in vivo* despite low titres of neutralising antibodies, where wild‐type adeno‐associated vectors are neutralised by humoral immunity.

### 4.3 Selecting the specific exosome parent cell

The origin of the exosome‐producing cells influence the exosome biodistribution and its therapeutic effect. Tumour‐derived exosomes have preferential tropism towards their parent cell type allowing a direct application in their native form to target these tumour cells (Sun et al., 2018). Specific engineering with expression of integrin β4 on the surface of MDA‐MB‐231 breast cancer cell‐derived exosomes provides preferential tropism and higher cellular uptake by non‐small cell lung cancer cells through interaction with their surfactant protein C (Nie et al., 2020). Furthermore, tumour‐derived exosomes can counteract some of the limitations in current immunotherapies and can be used to mediate immunosuppression in the tumour microenvironment by activating lymphocytes and dendritic cells (Sun et al., 2018). The self‐tolerance developed in tumour microenvironment overtime dampens the therapeutic effect of T cell responses against tumours, but tumour‐derived exosomes can induce CD8^+^ T cell suppressors against tumours (Maybruck et al., 2017), increase regulatory T cells resistance to apoptosis and up‐regulate their suppressor functions (Szajnik et al., 2010).

Similarly, mesenchymal stem cell‐derived exosomes can exhibit immunomodulation abilities and a strong tumour tropism. Increased expression of TNF‐related apoptosis‐inducing ligand (TRAIL) is observed in mesenchymal stem cell‐derived exosomes, which after interaction with death receptors 4/5 (DR4/TNFRSF10A: DR5/TNFRSF10B) mediates apoptosis specifically in cancer cells (Yuan et al., 2017). Exosomes derived from human umbilical cord‐ mesenchymal stem cells improve inflammation in carbon tetrachoide (CCl4)‐induced fibrosis in mouse livers (Li et al., 2013). Mesenchymal stem cell‐derived exosomes overexpressing GATA4 alleviate sequelae of ischemic heart disease via the anti‐apoptotic miRNA, miR19a, which promotes mesenchymal stem cell differentiation in cardiomyocytes, reduces cardiomyocyte apoptosis and enhances angiogenesis (Yu et al., 2015).

As part of their physiological function, exosomes are involved in the regulation of the immune system. Such exosomes derived from macrophages, dendritic cells and natural killer (NK) cells exhibit therapeutic effect by activating T cell responses and displaying anti‐tumour effects *in vitro* (Andaloussi et al., 2013; Quah & O'Neill, 2005). For example, NK‐derived exosomes primed with interleukin (IL)‐15 show specific tropism towards human cancer cells but can also trigger a cytotoxic effect (Zhu et al., 2019). Immune cell‐derived exosomes can also have an immunosuppressive role where regulatory T cells‐derived exosomes can inhibit the dendritic cell immune function via exosomal miRNA like miR‐150‐p and miR‐142‐3p (Tung et al., 2018). Similarly, exosomes from activated CD8^+^ T cells can suppress dendritic cell activation through the major histocompatibility complex (MHC) and via induction of apoptosis (Xie et al., 2010).

## 5 MANUFACTURING STRATEGIES AND LIMITATIONS

Exosome are defined by their cells of origin and their engineered properties. However, their efficient and reliable production is controlled by their microenvironment, culture and dissociation systems. Further downstream steps in their manufacturing are also required involving isolation and characterisation. This process is generally divided into three steps:‐ (i) removing of cells and cell debris, (ii) concentration of condition medium and (iii) purification (Chen et al., 2020; Gao et al., 2020).

Various types of cells have been used for good manufacturing practice exosome manufacturing such as HEK293 (Watson et al., 2018), mesenchymal stem cells (Pachler et al., 2017), dendritic cells (Lamparski et al., 2002), adipose tissue‐derived stem cells (Bari et al., 2019) and human cardiac progenitors cells (Andriolo et al., 2018). Those cell types might require specific culture parameters such as the need for growth factors, oxygen requirements, cell density, cell passage and cell differentiation (Panchalingam et al., 2015; Patel et al., 2018; Sart et al., 2010). The cultivation medium is usually defined into animal‐free (Andriolo et al., 2018; Lamparski et al., 2002) or animal‐derived (Mendt et al., 2018) components and is based on the type of cells used. However, for ethical issues and risks of contamination, animal‐derived components should be avoided. The use of xeno‐free conditions in good manufacturing practice production should be favoured as alternatives, xeno‐free media has also been shown to increase yield (Andriolo et al., 2018). The careful selection of a clone for further expansion is an option of choice: the clonal cell line will enable a homogeneous production and simplify the downstream characterisation process.

Culture systems include either static systems such as flasks, but scaling up processes will include dynamic systems like bioreactors which offer the advantage of improved controls of parameters such as CO_2_, O_2_ and pH. Microcarriers in stirred‐tanked bioreactors provide maximum surface area (Sart et al., 2010) and hollow fibre perfusion bioreactors (Yan & Wu, 2020) are also showing promising results. Hollow fibre bioreactors (Watson et al., 2016) tend to be used for conditioned medium harvest (Mendt et al., 2018; Watson et al., 2018). They also allow a more efficient production of exosomes compared with a static system. Dissociation enzymes if considered should be animal free (Andriolo et al., 2018).

Downstream processing involves differential centrifugation, the most common method for media concentration. Exosome purification methods rely on density centrifugation, precipitation, chromatography, membrane filtration and size exclusion. Each method will influence the amount, type and purity of exosomes (Yang et al., 2019). Tangential flow filtration is another method recently developed for media concentration and exosome isolation and is proven to be more gentle, efficient and scalable than ultracentrifugation and resulting in higher yield. It combines membrane filtration and a tangential flow across the surface to avoid filter cake formation (Busatto et al., 2018).

**FIGURE 3:**
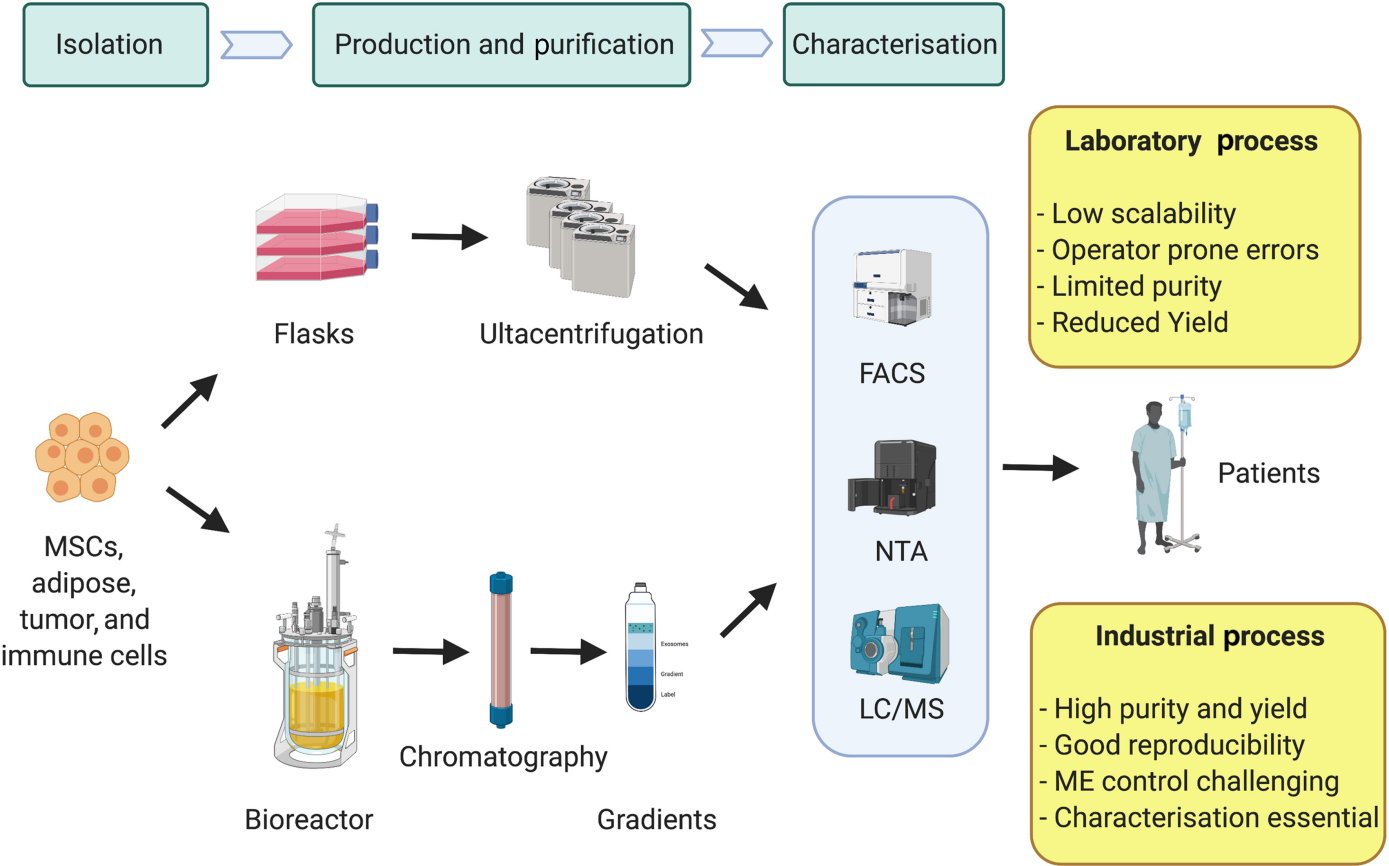
Exosomes manufacturing for clinical use: from the laboratory to the industry. Schematic highlighting current differences between academic and industrial scale up. Exosomes can be derived from a cell bank or from the patients themselves. Laboratory set up predominantly uses a cell flask platform while the scaling up process involves bioreactors. While bioreactors increase yields and purity, exosomes need to be adequately characterised. FACS: fluorescence‐activated cell sorting, LC/MS: liquid chromatography‐mass spectrometry, MSC: mesenchymal stem cells, ME: microenvironment, NTA: nanoparticle tracking analysis

As clinical applications of exosomes expand, manufacturing strategies initially developed in academic settings need to be adapted for scaling up (Figure 3) to meet adequate Chemistry, Manufacturing and Controls (CMC) timelines and good manufacturing practice requirements. However, this scale‐up process might alter the cell line phenotype and the downstream biological function of exosomes. For instance, this could impact the cellular microenvironment by modifying the cellular physiology, pH, oxygen supply, media composition and supply of growth factors (Chen et al., 2019). Also, the use of primary or stem cell lines adds further complexity to the process (Cheng et al., 2017; Sart et al., 2010) as some cell lines like mesenchymal stem cells have slower growth and altered biology during the scale‐up process (Chen et al., 2011). Cell density, apoptotic blebs and impurities from cells undergoing cell death can further impact the exosome functionality (Bollini et al., 2013; Crescitelli et al., 2013; Patel et al., 2018). Scaling up using bioreactors can also have mechanical consequences. mesenchymal stem cells, which have accrued potential for exosome production, can modify their cellular phenotype when exposed to shear stress in a bioreactor (Brindley et al., 2011; Panchalingam et al., 2015). Cyclic stretch combined with shear stress inhibits their smooth muscle actin formation and induces a switch of mesenchymal stem cell phenotype towards endothelial cells (Patel et al., 2018). An alternative to bioreactor is to use multiple flasks or stacked multilayer culture flasks, but this may not be a compatible option for industrial scale‐up. One popular and cost‐effective system is the Integra CELLine™ system, which allows high yield of exosomes compared to flasks without altering the exosome morphology, phenotype and function (Mitchell et al., 2008). The CELLine™ system uses different compartments for cells and media allowing a continuous flow of nutrients while reducing waste products and allowing optimal cell proliferation. Another limiting factor for scaling up is the need of serum and growth factors in large volumes (Shelke et al., 2014). Sera like fetal bovine serum are rich in endogenous exosomes and contaminants, a major pitfall for good manufacturing practice production. Furthermore, the drug‐loading efficacy in exosomes is influenced by the production cell line, the cargo and the loading method and can vary from 1.4% to 38% (Walker et al., 2019).

As extracellular vesicles are heterogeneous by nature, the purification process is critical, which limits the downstream scale‐up. Purification is frequently achieved using operator‐dependent ultracentrifugation which produces low yields (Lee, Johansson, et al., 2019). However, this challenge is progressively being overcome as illustrated by the production of good manufacturing practice‐compliant mesenchymal stem cells‐derived exosomes for clinical trials (Chen et al., 2019). Additional purification steps are often required which include exclusion size chromatography and sucrose or Optiprep™ density gradients. Physical modification with the formation of exosome aggregates is also a significant drawback of ultracentrifugation as few reports suggest the exosome original phenotype and morphology to be compromised causing artefacts or undesired effects (Linares et al., 2015). An adaptation of the rotor size and type (Cvjetkovic et al., 2014) can partly alleviate these detrimental effects. Combined or as an alternative to ultracentrifugation, filtration and size exclusion chromatography methods are also commonly used for isolation. A promising technique combining bind‐elution with size exclusion chromatography shows high yields and better reproducibility. This technology enables elution of large particles like exosomes while smaller impurities like nonvesicular proteins and RNAs remain bound to the column. It is highly reproducible generating up to 80% yields even when used with large volumes of media (Corso et al., 2017) while limiting risks of vesicular disruption and aggregation.

A safety regulatory framework with good manufacturing practice standards for exosome characterisation throughout the manufacturing process is being developed by the International Society for Extracellular Vesicles or the European Network on Macrovesicles and Exosomes in Health and Disease (Lener, Gimona, Aigner, Börger, Buzas, Camussi, Chaput, Chatterjee, Court, Portillo, & O'Driscoll, 2015a). This standardisation is paramount for the clinical translation of exosomes (Ilic et al., 2012; Witwer et al., 2013). Exosome characterisation includes (i) protein quantification using techniques such as bicinchoninic acid (BCA) assay, western blotting or liquid chromatography and mass spectrometry for proteomic analysis (Yang, Guo, et al., 2017), size and concentration assessed by nanoparticle tracking analysis (Dragovic et al., 2011), (ii) morphology by transmission electron microscopy (Chuo et al., 2018) and (iii) exosome surface profiling or cargo characterisation by flow cytometry and liquid chromatography or mass spectrometry (Schey et al., 2015).

Finally, standardising storage methods of pure extracellular vesicle fractions is also a crucial step for translation (Mora et al., 2015) (Lener, Gimona, Aigner, Börger, Buzas, Camussi, Chaput, Chatterjee, Court, Portillo, O'Driscoll, Fais, et al., 2015b). Storage at 4°C or −80°C can impact the biological activity and protein content of exosomes (Maroto et al., 2017). Storage at −80°C is advised as the optimal temperature providing the least impact on exosome morphology and content (Yamashita et al., 2018). Other parameters adding to the complexity of the scale‐up process are the storage buffer and its pH, the number of freeze–thaw cycles and their effect on exosomal protein content (Cheng et al., 2019).

## 6 CONCLUSION

Exosomes are poised at crossroads for clinical applications. The use of these small vesicles is rapidly expanding for diagnostic and therapeutic purposes following their relative recent discovery as key players in physiology and pathology. The leading clinical applications in cancer and inflammation are exploiting the exosome role in immunomodulation and its use as a vehicle for specific drug delivery. Numerous academic publications as well as high value investments in pharmaceutic development dedicated to the exosome field demonstrate general and cross‐sector enthusiasm for these biotechnologies. Various strategies to optimise the therapeutic efficacy of exosomes are being developed. The regulatory framework is evolving in order to allow safe and successful clinical trials. Improved scaling up strategies are being developed in order to overcome the limitations of manufacturing and characterisation processes. Hence, while still at its infancy, the exosome field is moving fast to maturity for the benefit of the patients.

### 6.1 Nomenclature of targets and ligands

Key protein targets and ligands in this article are hyperlinked to corresponding entries in the IUPHAR/BPS Guide to PHARMACOLOGY http://www.guidetopharmacology.org and are permanently archived in the Concise Guide to PHARMACOLOGY 2019/20 (Alexander et al., 2019)

## AUTHOR CONTRIBUTIONS

D.P. and J.B. designed the work. D.P. and L.T. wrote the manuscript. All authors revised substantially and approved the final manuscript. All authors accept full responsibility of its content.

## CONFLICT OF INTEREST

The authors are partially funded by a United Kingdom Research Council Innovate UK Biomedical Catalyst award (14720) awarded jointly with Evox Therapeutics.
